# Elevated triglyceride–glucose index increases risk in patients with retinal vein occlusion

**DOI:** 10.3389/fmed.2025.1702069

**Published:** 2026-01-09

**Authors:** Bingyang Lv, Kaichao Xia, Yang Yang, Ziyan Song, Yiqiao Xing, Anhuai Yang, Kaibao Ji

**Affiliations:** Department of Ophthalmology, Renmin Hospital of Wuhan University, Wuhan, Hubei, China

**Keywords:** atherosclerosis, retinal vascular disorder, retinal vein occlusion, risk factor, triglyceride-glucose index

## Abstract

**Background:**

Retinal vein occlusion (RVO) is the second most prevalent retinal vascular disorder, and persistent macular edema secondary to RVO is a leading cause of visual impairment. Emerging studies have indicated that the triglyceride–glucose (TyG) index holds clinical significance in assessing vascular damage. This study aimed to investigate the role of the TyG index in patients with RVO.

**Methods:**

In this retrospective study, we examined 51 patients diagnosed with RVO alongside 54 age- and sex-matched control subjects. Comprehensive ocular examinations were performed, and various hematological parameters, including fasting blood glucose, total cholesterol, triglycerides, high-density lipoprotein cholesterol, and low-density lipoprotein cholesterol, were obtained through blood biochemistry tests. The TyG index was calculated using fasting plasma glucose and triglyceride levels. A logistic regression analysis was used to assess the association between these lipid markers and the risk of RVO. The receiver operating characteristic (ROC) curve was used to evaluate the predictive values of the TyG index in relation to RVO. Data analysis was conducted using IBM SPSS Statistics (Version 22), with statistical significance set at a *p*-value of < 0.05.

**Results:**

Among the hematological parameters assessed, no statistically significant differences were observed between the two groups regarding glucose, total cholesterol, and low-density lipoprotein cholesterol (LDL-C) (*p* > 0.05). However, triglyceride levels in the RVO group were significantly increased compared to the control group (*p* < 0.0001). Additionally, TyG values were markedly higher in individuals with RVO than in controls (*p* < 0.0001). In a multivariate logistic regression analysis, the TyG index was significantly associated with RVO after adjusting for age and sex (OR = 48.21; 95% CI: 7.19–323.41; *p* < 0.001). Notably, the TyG index showed an AUC value of 0.819, along with favorable sensitivity (72.55%) and high specificity (83.33%), suggesting its potential as a promising biomarker for both the diagnosis and prognosis of RVO.

**Conclusion:**

Our findings indicate that the TyG index is elevated in patients diagnosed with retinal vein occlusion. Therefore, the TyG index may serve as a significant predictive biomarker for identifying individuals at risk of developing retinal vein occlusion.

## Introduction

Retinal vein occlusion (RVO) ranks as the second most common retinal vascular disease following diabetic retinopathy and is recognized as a significant contributor to visual impairment (1). Based on occlusion location, RVO can be subdivided into central retinal vein occlusion (CRVO) with a prevalence of 0.1–0.4% and branch retinal vein occlusion (BRVO) affecting 0.6 to 1.6% (2). It is estimated that approximately 16 million individuals worldwide are affected by RVO, with BRVO accounting for approximately four-fifths of cases (3). Various risk factors, including systemic arterial hypertension, hypercholesterolemia, diabetes mellitus, smoking habits, increased body mass index (BMI), thrombophilia and hypercoagulation, ocular factors such as intraocular hypertension or glaucoma, and orbital diseases, contribute to this condition (4–9). The precise pathophysiology of RVO remains incompletely understood. Previous studies have indicated that compression and mechanical stenosis of the retinal vein, resulting from an atherosclerotic artery, along with local inflammation triggered by venous stasis and exudation, represent the primary pathogenetic mechanisms in the majority of cases (10, 11). In RVO, two primary pathophysiological pathways are identified: (1) damage to the inner blood–retinal barrier facilitated by the release of inflammatory cytokines and reactive oxygen species (ROS) generated from oxidative stress and (2) tissue hypoxia resulting from vascular closure, which leads to an increased expression of vascular endothelial growth factor (VEGF) (12). Additionally, individuals with systemic arteriosclerotic vascular disease are at an elevated risk of developing this condition (13).

The triglyceride–glucose (TyG) index has gained recognition in recent years as a reliable biomarker for insulin resistance. Several studies have demonstrated an association between the TyG index and conditions such as ischemic stroke, hypertension, and cerebrovascular disease (14–16). Furthermore, a strong correlation exists between the TyG index and cardiovascular diseases related to atherosclerosis (17). Research indicates that a high TyG index is linked to increased arterial stiffness, highlighting its clinical significance in assessing microvascular damage (18). Previous investigations have shown that RVO shares similar pathological mechanisms and risk factors with cardiovascular diseases (19).

Although emerging research has explored the relationship between the TyG index and BRVO (20), available data remain limited, leaving the precise role of the TyG index in RVO inadequately defined. Therefore, the objective of the present study is to investigate the role of the TyG index in patients diagnosed with RVO.

## Methods

### Study population

This retrospective case–control study was performed in accordance with the principles outlined in the Declaration of Helsinki and received approval from the Ethics Committee of Renmin Hospital of Wuhan University. Digital medical records of patients diagnosed with RVO and the control group between May 2023 and January 2024 were collected and analyzed. A total of 51 patients diagnosed with RVO and 54 age-sex-matched healthy individuals, who exhibited comparable risk factors for systemic disorders such as hypertension and diabetes mellitus, were included in the study. The diagnoses of retinal vein occlusion were performed as previously outlined (21), encompassing flame-shaped and dot-blot hemorrhages, edema, cotton-wool spots, hard exudates, venous dilation, and tortuosity. The control group comprised healthy individuals who were admitted to the ophthalmology outpatient clinic for routine examinations.

All participants underwent a thorough ocular examination, which included best-corrected visual acuity (BCVA) assessment, intraocular pressure (IOP) measurement, slit-lamp biomicroscopy, fundoscopic evaluation following pupil dilation, spectral-domain optical coherence tomography (SD-OCT), and fundus fluorescein angiography (FFA).

The exclusion criteria were participants with uncontrolled diabetes mellitus and hypertension, cardiovascular and cerebrovascular diseases, acute or chronic renal failure, acute or chronic hepatic disorders, chronic obstructive pulmonary disease (COPD), anemia, malignant tumors, chronic smoking habits, alcohol abuse, acute infectious diseases, and chronic systemic inflammatory conditions. Participants with ocular pathologies such as prior ocular surgery, trauma to the eye, ocular infections, uveitis, scleritis, or retinal vasculitis were also excluded.

### Laboratory assessment

All peripheral venous blood samples were collected after an 8–12 h overnight fast on the second day following the initial diagnosis of RVO. Comprehensive blood biochemistry analyses were conducted using an automatic biochemical analyzer (ADVIA® Chemistry XPT System, Tokyo, Japan). The levels of fasting blood glucose (FBG), total cholesterol (TC), triglycerides (TG), high-density lipoprotein cholesterol (HDL-C), and low-density lipoprotein cholesterol (LDL-C) were quantified. The TyG index was calculated using the formula ln [fasting triglycerides (mg/dL) × fasting glucose (mg/dL)/2] (22). Two investigators (Bingyang Lv and Kaichao Xia) independently extracted relevant data from the selected records, with discrepancies resolved through discussion or consultation with the third author (Yang Yang). The following information was extracted: age; sex; presence or absence of hypertension; diabetes mellitus; and primary hematological parameters, including glucose, total cholesterol, triglycerides, HDL-C, LDL-C, and TyG index.

### Statistical analysis

Statistical analysis was conducted utilizing IBM SPSS Statistics (Version 22). Normal distribution of data was assessed using the Shapiro–Wilk test. Normally distributed continuous data are presented as mean ± standard deviation (SD), while non-normally distributed data are reported as median and interquartile range (IQR). Continuous variables between groups were compared using an independent samples t-test or the Mann–Whitney U-test, depending on their distribution characteristics. Categorical variables are expressed as numbers and percentages, with group differences evaluated using the chi-squared test. A logistic regression analysis was used to identify independent predictors of RVO. Receiver operating characteristic (ROC) curve analysis was performed to ascertain the cutoff threshold and evaluate the accuracy of these indicators. The optimal cutoff value was determined using the Youden index. A two-tailed *p*-value of less than 0.05 was deemed statistically significant.

## Results

A total of 105 participants were enrolled in this study, comprising 51 individuals in the RVO group and 54 in the control group. The median age of participants in the RVO group (27 women and 24 men) was 59.00 years (range: 52.00 to 69.50), while that of the control group (23 women and 31 men) was 58.00 years (range: 55.00 to 64.75). No significant differences were observed between the groups regarding age and sex distribution (*p* = 0.488 and *p* = 0.289, respectively). Table 1 presents the basic characteristics and laboratory measurements pertinent to this study.

**Table 1:** Demographic and hematological characteristics of the control and RVO groups are presented.

Variables	Control	RVO	*p*-value
Age (years)	58.00 (55.00, 64.75)	59.00 (52.00, 69.50)	0.488^a^
Sex (Female/Male)	23/31	27/24	0.289^b^
Hypertension (*n*)	24	26	0.503^b^
Diabetes mellitus (*n*)	4	5	0.929^b^
Glucose	4.75 (4.49, 5.27)	5.02 (4.73, 5.36)	0.056^a^
Total cholesterol	4.58 (4.28, 4.88)	4.61 (3.97, 5.06)	0.840^a^
Triglycerides	1.02 (0.84, 1.18)	1.53 (1.12, 1.92)	< 0.0001^a^
HDL-C	1.26 (1.14, 1.34)	1.09 (0.97, 1.18)	< 0.0001^a^
LDL-C	2.69 (2.33, 3.02)	2.78 (2.07, 3.11)	0.847^a^
TyG index	8.33 (8.05, 8.44)	8.77 (8.45, 9.00)	< 0.0001^a^

Data are presented as median and interquartile range or frequency, as appropriate. Comparisons between groups were performed using the Mann–Whitney U-test for continuous variables or the chi-squared test for categorical variables. A *p*-value of < 0.05 was considered statistically significant. RVO, retinal vein occlusion; HDL-C, high-density lipoprotein cholesterol; LDL-C, low-density lipoprotein cholesterol; TyG index, triglyceride–glucose index. “^a^” refers to the Mann-Whitney U test. “^b^” denotes the Chi-Square test.

**Figure 1:**
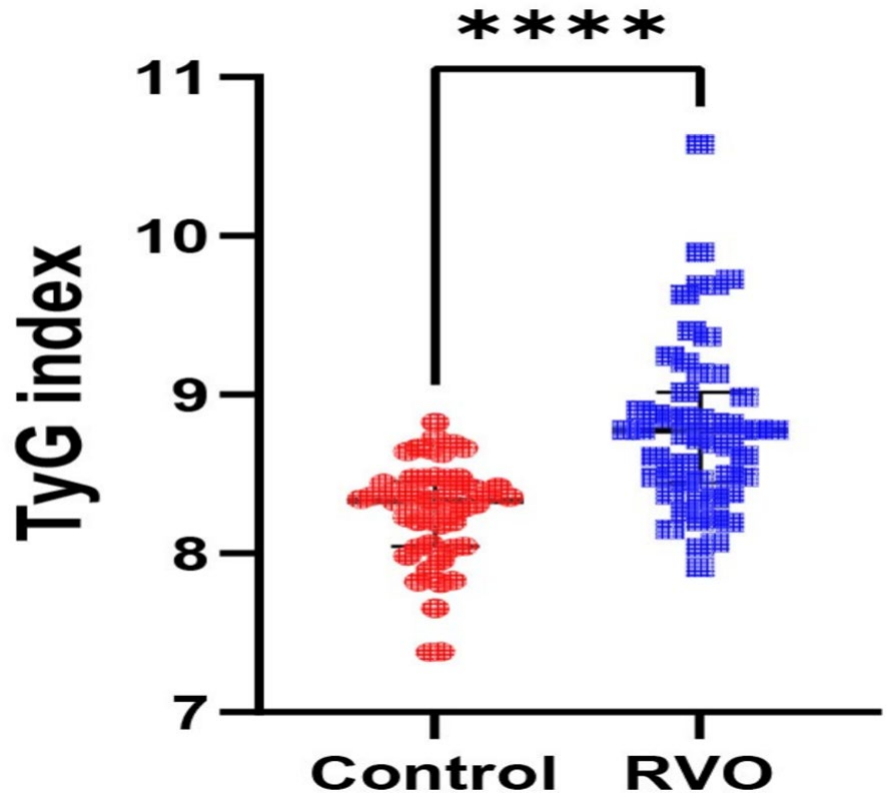
Comparison of the TyG index was made between the control group and the RVO group. Data are reported as median and interquartile range. Comparison between groups was conducted using the Mann–Whitney *U*-test, and a *p*-value of < 0.05 was considered statistically significant. TyG index, triglyceride–glucose index; RVO, retinal vein occlusion, ****p < 0.0001.

Upon comparison between the RVO group and the control group, no statistically significant differences were found in terms of hypertension (*p* = 0.503) or diabetes mellitus (*p* = 0.929). Examination of laboratory parameters revealed no remarkable differences between the two groups for blood glucose levels (*p* = 0.056), TC levels (*p* = 0.840), or LDL-C levels (*p* = 0.847). However, TG values were significantly higher in the RVO group compared to the control group (*p* < 0.0001), and HDL-C levels were remarkably lower in the RVO group than in the control group (*p* < 0.0001). Additionally, there was a substantial increase in TyG index values within the RVO group relative to the control group (p < 0.0001), as described in Figure 1.

A multivariate logistic regression analysis was conducted to predict the independent risk factors in discriminating between the RVO and control groups, and the TyG index (OR = 48.21; 95%CI: 7.19–323.41; *p* < 0.001) was found to be associated with RVO (Table 2). Based on the receiver operating characteristic curve analysis, it was found that the area under the ROC curve of the TyG index for predicting patients with RVO was 0.819.

**Table 2:** Predictors of RVO were identified in multivariate regression analysis.

Variables	OR (95% CI)	*p*-value
TyG index	48.21 (7.19 ~ 323.41)	< 0.001
Age	1.01 (0.98 ~ 1.05)	0.472
Sex (female)	1.36 (0.34 ~ 5.37)	0.662

Covariates included age, sex, and TyG index. OR, odds ratio; CI, confidence interval; TyG index, triglyceride–glucose index. A *p*-value of < 0.05 was deemed statistically significant.

**Figure 2:**
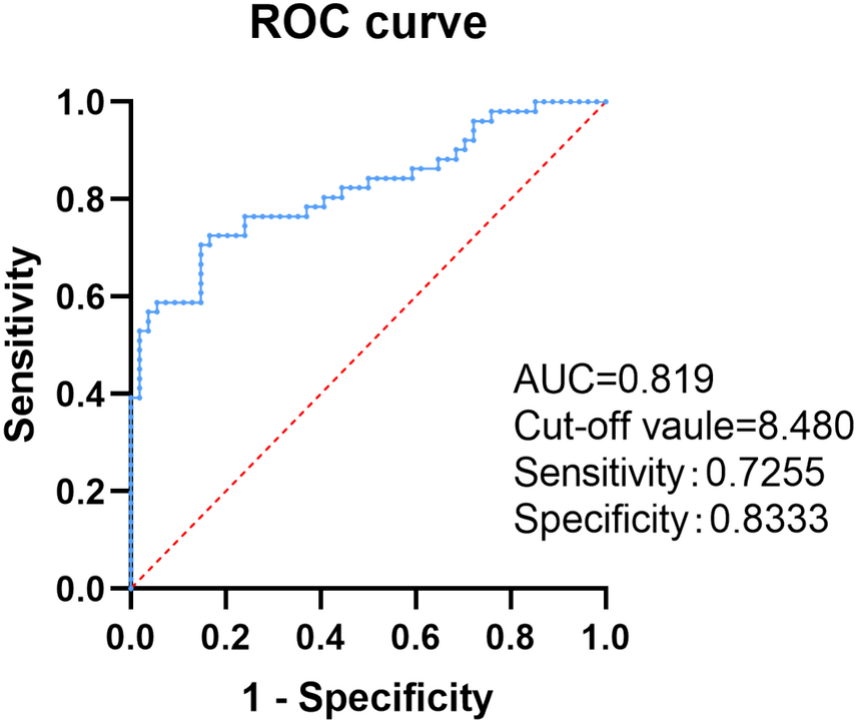
Receiver operating characteristic curve analysis of the TyG index for predicting retinal vein occlusion. TyG index, triglyceride–glucose index; ROC, receiver operating characteristic, and AUC, area under the curve.

The optimal cutoff point for the TyG index was 8.480, with a sensitivity of 72.55% and a specificity of 83.33%, as shown in Figure 2.

## Discussion

In the current study, we explored the TyG index, a recently identified biomarker for atherosclerosis, in patients with retinal vein occlusion. Compared to the healthy control group, patients with RVO exhibited significantly higher TyG levels. A high TyG index was associated with an elevated risk of RVO. Additionally, based on the analysis of the receiver operating characteristic curve, we discovered that the TyG index serves as a convenient predictor of the disease.

RVO is globally acknowledged as a serious retinal vascular disorder, potentially causing irreversible vision loss if not treated promptly. Although the pathogenic mechanism of RVO is multifactorial and its precise pathophysiology remains unclear, atherosclerosis and inflammation are widely recognized as crucial factors in its development. Several risk factors, including arterial hypertension, dyslipidemia, and diabetes mellitus, have been associated with the development of RVO. Hypertension is regarded as the most potent risk factor since it accelerates arterial atherosclerosis, thereby causing mechanical compression of adjacent venous vessels (12). This sequence of events leads to mechanical lumen narrowing of the thin-walled veins, abnormal blood flow, and endothelial damage, resulting in the formation of endoluminal thrombus and occlusion (12). Janssen et al. (23) have reported that several thrombotic risk factors, such as hyperhomocysteinemia, MTHFR gene mutation, antiphospholipid antibodies (APL), and lipoprotein (a) [Lp(a)], were demonstrated to be independent risk factors for RVO. Apart from atherosclerosis, inflammation induced by venous stasis is another predominant pathogenetic event in most cases of RVO. Previous studies have indicated that levels of cytokines, such as VEGF, interleukin (IL)-6 and 8, monocyte chemotactic protein (MCP)-1, and platelet-derived growth factor (PDGF)-AA, are associated with increased vascular permeability (24, 25). Vascular endothelial injury, venous stasis, and hypercoagulable states are involved in thrombogenesis, resulting in RVO (26).

Retinal vascular diseases and cardiovascular disorders share common atherosclerotic mechanisms and risk factors (27). The TyG index is significantly associated with the risk of atherosclerotic cardiovascular diseases (17). Luo et al. (28) have demonstrated that an elevated TyG index value is correlated with an increased risk of major cardiac and cerebrovascular events. Moreover, a previous study found that a high TyG index was also strongly linked to symptomatic coronary artery disease and that this parameter could be used as a marker of atherosclerosis (29). In the present study, our data indicated that the TyG index values were remarkably higher in the RVO group than in the control group, suggesting that the TyG index is a potential risk factor for RVO and may contribute to its development by promoting atherosclerotic events. Katipoğlu and Turan (30) reported that RVO patients had a significantly higher TyG index compared to the controls, consistent with our findings. Additionally, a previous study (20) comparing BRVO patients with controls found that the TyG index was significantly elevated in the BRVO group. Therefore, the increased TyG index observed in RVO patients suggests that this parameter may be associated with the development and clinical outcomes of RVO. However, both studies (20, 30) are observational in nature and thus unable to establish a causal relationship between the TyG index and RVO. Large-scale prospective cohort studies are needed to confirm the association. Apart from the TyG index, calculated as ln [fasting triglycerides (mg/dL) × fasting glucose (mg/dL)/2], lipid ratios offer a comprehensive assessment of both atherogenic and antiatherogenic lipid components (31). Evidence from previous studies indicates that lipoprotein ratios—such as TG/HDL-C, TC/HDL-C, LDL-C/HDL-C, and non-HDL-C/HDL-C—demonstrate stronger associations with the severity and prevalence of coronary artery disease than individual lipid parameters, thereby providing enhanced utility in evaluating atherosclerosis risk (32). A recent study found that patients with RVO exhibited significantly higher levels of TG/HDL-C, LDL-C/HDL-C, TC/HDL-C, and non-HDL-C/HDL-C compared to healthy controls (33). The homeostasis model assessment of insulin resistance (HOMA-IR), calculated from fasting plasma glucose (FPG) and insulin levels using the method described by Matthews et al. (34), has been used in previous studies, which reported a positive correlation between the TG/HDL-C ratio and HOMA-IR in RVO patients (33). Furthermore, atherosclerosis is a well-documented risk factor for RVO, and dysregulation of cholesterol metabolism exacerbates this association (35). The LDL-C/HDL-C ratio is an established marker of ischemic heart disease, with elevated values indicating impaired cholesterol metabolism (36). The TC/HDL-C ratio, which integrates both atherogenic and antiatherogenic lipids, serves as an independent predictor of coronary heart disease (37). Additionally, the non-HDL-C/HDL-C ratio has been shown to outperform individual lipid measures in identifying metabolic syndrome and insulin resistance (38). Elevated LDL-C/HDL-C, TC/HDL-C, and non-HDL-C/HDL-C ratios are linked to RVO and may function as valuable biomarkers for its development. Therefore, future research should explore the relationships between these lipid ratios and the TyG index to identify optimal predictive markers for RVO onset.

Although the possible mechanisms of the TyG index in relation to atherosclerosis have not yet been completely elucidated, several probable explanations have been put forward. First, a positive association between the TyG index and hypertension has been validated by a growing body of evidence from several cohort studies (39, 40), and a meta-analysis revealed that the TyG index can be used as a valuable predictor for identifying hypertension risk among the general adult population (41). Taking all of these studies together, hypertension, a major risk factor in RVO, significantly contributes to the occurrence of atherosclerosis. Second, the TyG index has been acknowledged as a reliable indicator of insulin resistance (42). A previous study disclosed its superior performance compared to the homeostatic model assessment for detecting insulin resistance (43), with high sensitivity (96.5%) and specificity (85.0%) (44). It has been hypothesized that insulin resistance is associated with persistent, low-grade systemic inflammation (45) and hyperglycemic damage (46). In addition, when triglyceride values are extremely high, the triglyceride-rich chyle particles are overly large to cross the endothelial barrier into the intima, thereby leading to atherosclerosis (47). Furthermore, insulin resistance may directly contribute to endothelial dysfunction, a key pathophysiological process in the initiation and progression of atherosclerosis (48). Calculated from two routine blood biochemical parameters (fasting triglycerides and fasting glucose), the TyG index is easily accessible and cost-effective. Based on our findings, the TyG index is a simple and potentially clinically useful marker for predicting the development of RVO in real-world clinical practice.

Some limitations should be taken into account in the present study. First, the sample size was relatively small, which may limit the applicability of our results to a larger population. Second, as a retrospective observational study, it can only establish temporal associations between exposure and disease and restrict the exploration of causality between the TyG index and RVO. Third, this was a single-center study including only Chinese participants, which may restrict the generalization of the results to other populations. Therefore, large-scale prospective cohort studies are needed to further validate the association between the TyG index and RVO.

## Conclusion

In summary, our results indicate that the TyG index is elevated in patients diagnosed with RVO. These findings provide clinical evidence that the TyG index could serve as a valuable biomarker for identifying populations at risk of developing RVO. Further large-scale prospective studies are warranted to investigate the mechanisms underlying the relationship between the TyG index and RVO.

## Data Availability

The original contributions presented in the study are included in the article/supplementary material, further inquiries can be directed to the corresponding authors.
